# Identification of Potential Inhibitors Targeting Non-Structural Proteins NS3 and NS5 of Dengue Virus Using Docking and Deep Learning Approaches

**DOI:** 10.3390/ph18040566

**Published:** 2025-04-13

**Authors:** Alomgir Hossain, Faria Tasnin Joti, Md. Shohag Hossain, Abdullah Al-Noman, Chomong Thowing, Mehjabin Mursona, Md. Robiul Islam, Md. Ekhtiar Rahman, Mohammad Nurul Matin, Md Azizul Haque

**Affiliations:** 1 Computational Biosciences and Chemistry Research Organization, Rajshahi 6205, Bangladesh; alamgir199817@gmail.com (A.H.); fariatasnin2020@gmail.com (F.T.J.); shohaghossain.ofc@gmail.com (M.S.H.); alnoman.ofc@gmail.com (A.A.-N.); chomong1614@gmail.com (C.T.); mehjabinmukto32@gmail.com (M.M.); s2111163134@ru.ac.bd (M.R.I.); ekhtiarbdj@gmail.com (M.E.R.); 2 Department of Genetic Engineering and Biotechnology, University of Rajshahi, Rajshahi 6205, Bangladesh; 3 Department of Biotechnology, Yeungnam University, Gyeongsan 38541, Republic of Korea

**Keywords:** dengue virus, NS3 and NS5 inhibitors, drug discovery, molecular docking, molecular dynamic simulation, deep learning

## Abstract

**Background**: Dengue virus (DENV) is the fatal pathogenic arthropod-borne virus (arboviruses) that belongs to the *Flaviviridae* family, which transmits to humans through mosquito bites from infected *Aedes aegypti* and *Aedes albopictus* mosquitoes or maternal-fetal transmission. Despite antigenic differences, the four serotypes of DENV (DENV-1 to DENV-4) share 65–78% of their genome. Non-structural (NS) proteins amongst serotypes show analogous functions. Among NS proteins, NS3 and NS5 are frequently used as targets for antiviral drugs due to their multifunctional roles. **Methods**: To identify potential inhibitors of DENV, we created a phytochemical library of 898 compounds derived from 17 medicinal plants recognized for their medicinal and antiviral properties. The phytochemicals library has been docked against the target proteins. Phytochemicals with a docking score greater than −8.0 kcal/mol were selected for further evaluation using a machine learning approach. Further, molecular dynamics (MD) simulations were conducted to evaluate the root mean square deviation, root mean square fluctuation, solvent-accessible surface area, radius of gyration, and hydrogen bond count of the compounds. **Results**: From the docking results, Silibinin, Rubiadin, and Ellagic acid showed binding affinities of −8.5, −8.3, and −8.2 kcal/mol, respectively, for NS3, and NSC 640467, Bisandrographolide A, and Andrographidin A showed binding affinities of −9.3, −10.1, and −9.3 kcal/mol, respectively, for NS5 target proteins. These compounds exhibited strong interactions with target proteins. MD simulation results confirmed the stable formation of protein–ligand complexes. Further, absorption, distribution, metabolism, excretion, and toxicity (ADMET) and bioactivity predictions confirmed their pharmacological safety. **Conclusions**: Despite global public health concerns, DENV still lacks specific drug treatments. Our identified new drug candidates might help for developing effective antiviral inhibitors against the DENV. However, further confirmation is needed through in vivo and in vitro research.

## 1. Introduction

Dengue fever is a mosquito-borne, viral disease signified by fever, headache, and arthralgia as well as dengue-shock syndrome [1], caused by Dengue virus [2], which has been a continual global health threat specifically in tropical and subtropical areas around the world [3,4]. The World Health Organization (WHO) estimated that dengue fever affects 100 million people annually, with half a million requiring hospitalization and approximately 25,000 cases resulting in fatal outcomes [5]. According to a recent study, there are 390 million dengue infections annually, of which 96 million present clinical symptoms worldwide, and about half the world’s population is now at risk, which is more than three times as many cases as the WHO reports [6]. Dengue fever is endemic in over 100 countries, primarily in Asia, Africa, the Pacific and Gulf coastlines of the United States, and the Caribbean until early this century [7]. DENV, an RNA arbovirus [8], belongs to the *Flaviviridae* family, which consists of 70 other viruses including yellow fever virus, Zika virus, Japanese encephalitis virus, and West Nile virus [9]. DENV can be transmitted to humans through mosquito bites from infected *Aedes aegypti* and *Aedes albopictus* [4,10], and through the maternal–fetal mode of transmission [11]. Despite having different antigens, DENV serotypes share 65% of their genomes [12]. When an infection is caused by one serotype, it does not provide cross-protection against subsequent infections caused by other serotypes. All of the serotypes can induce coagulopathy, increased vascular fragility and permeability, and mild to severe symptomatic dengue fever, followed by a disorder named dengue shock syndrome (hypovolemic shock) [11].

The genome of DENV comprises a positive-sense RNA of 11 kb in length, which encodes a polyprotein precursor weighing around 370 kDa, which includes the structural proteins C, prM, and E, along with non-structural proteins designated as NS1, NS2A, NS2B, NS3, NS4A, NS4B, and NS5. The proteases of host cells, along with the viral protease NS3, cleave the polyprotein. This process generates structural proteins, like capsid, pre-membrane, and envelope protein, which form virion, as well as non-structural proteins essential for viral maturation and replication [13,14]. The NS2A/NS2B, NS2B/NS3, NS3/NS4A, NS4B/NS5, and capsid protein are all cleaved by the NS2B–NS3 protease complex that is encoded by the virus [15]. The widely studied structural protein is the envelope protein, which has various roles to play from viral entrance to RNA release into the cytoplasm, whereas NS3 and NS5 are frequently used as the targets of antivirals due to their multifunctional activities [16].

The second biggest non-structural DENV protein, NS3 protease, can act as a two-edged blade with its N-terminal protease (PRO), C-terminal helicase (HEL) activities in addition to 5′ RNA triphosphatase (5′ RTP) activities, that are involved in the RNA capping process [17]. The crystal structure of NS3 reveals a traditional chemo-trypsin-like fold featuring two β-barrels at the N-terminal and RNA tri-phosphatase and helicase domains at the C-terminal. At the clef of two beta-barrels, a conserved catalytic triad (His51–Asp75–Ser135) shows major functional importance. For ideal enzymatic activity, the beta-barrel region of NS3 makes chimera with the NS2B (49–95 aa) at the hydrophilic part and functions as a cofactor by shielding NS3 hydrophobic residues [18]. Functional disruption of the NS3/NS2B complex resulted in inhibition of viral replication and infectivity. In BHK-21 mammalian cells, viral replication was inhibited when NS3 full-length, PRO, or HEL region was mutated [17], suggesting NS3 potentially interacts with DENV proteins. Therefore, the NS3/NS2B has been identified as a suitable target protein for screening and assessing the effects of various drug candidates [19,20,21].

The NS5 protein is the largest and conserved protein in flaviviruses, consisting of around 900 amino acids and weighing approximately 100 kDa. It features an RNA-dependent RNA polymerase (RdRp) domain at its C-terminus, which is essential for viral replication [22,23]. This large protein also contains a capping enzyme site consisting of a methyltransferase (MTase) domain and a guanylyltransferase (GTase) domain at the N-terminus [24,25,26]. The NS5 capping enzyme plays an essential role in mRNA capping due to its methyltransferase and guanylyltransferase activities [27]. The N-terminus of NS5-CE is responsible for adding methyl groups to the guanine N-7 and ribose 2′ hydroxyl positions of the viral cap [28,29]. While the NS5 protein is primarily involved in viral replication within the cytoplasm of infected host cells, it predominantly localizes to the nucleus, indicating its potential role in suppressing the host’s antiviral response [30]. The enzymatic activity of NS5 indicates its important role in viral replication, as this enzyme plays a key role in maintaining viral lifespan and viability, making NS5 a promising antiviral target [31]. Recent identification showed that NS3 and NS5 inhibited the interaction between DENV NS3 and NS5 and the replication of all DENV serotypes and exhibited low propensity [32], suggesting that targeting NS3–NS5 interaction is a potential therapeutic approach that can lower virus replication and show novel chemical scaffolds to be developed into broad-spectrum antiflaviviral drugs [17,32].

There is currently no antiviral drug available to treat DENV [33]. The current treatments just centered on treating the indications, depending totally on supportive care [34]. In emergencies, the bioburden of viral load may be decreased with the use of antiviral medicines. The only FDA-approved intervention is the Dengvaxia vaccine, which is restricted to individuals aged 9–16 with prior dengue infection in endemic regions. Qdenga, another vaccine, is approved in some countries but not by the FDA. Constant mutations in a viral genome led to changes in the binding site polymorphisms and a decrease in affinity. For this reason, traditional antiviral drugs are insufficient in curing and restoring the disease [35]. Therefore, in the event of dengue viral infestations, the urgent need for alternatives is crucial. Several repurposed medications, such as sinefungin, ivermectin, lovastatin, prednisolone, modipafant, ketotifen, favipiravir, ribavirin, celgosivir, UV-4B, and chloroquine, have been tested against dengue virus, but no significant results have been recorded. Additionally their safety issues, side effects, and toxicity are still not beyond the controversy [11]. Moreover, infection with one serotype provides lifelong immunity to that serotype but not to others, making patients susceptible to multiple infections for a lifetime. Therefore, understanding each serotype is a prerequisite to identifying antibodies specific to the virus. Although characteristics of DENV are well-defined, they are still unpredictable with uncommon clinical manifestations, amazing transmission, persistency, and the discovery of new species. On the other hand, natural products are an enriched source of medicines for dengue fever [4]. Therefore, the search for novel anti-dengue agents from phytochemicals is highly emergent now. Phytochemicals are plant-derived natural products that have been recognized as effective and beneficial substitutes to manage numerous infections such as dengue [36]. This demand is based on medicines that are safe, less harmful than synthetic drugs, as well as non-toxic.

The purpose of this study is to screen a large library of phytochemicals that may be able to inhibit various DENV serotypes by taking advantage of their conserved proteases. Using a multi-target strategy, the study used virtual ligand screening to identify a group of phytochemicals that may selectively hinder the entire spectrum of DENV serotypes and inhibit highly conserved non-structural viral proteins. The study concentrated on targeting the nonstructural DENV proteins NS3/NS2B, and NS5, which are in charge of viral replication and the DENV antibody-dependent enhancement (ADE) phenomena, to achieve that goal. Using molecular dynamics (MD) simulations and a structure-based drug design technique, various phytochemicals were virtually tested against the previously identified NS proteins of DENV. Furthermore, these approaches offered a deeper understanding of the structural characteristics and mechanisms governing the interactions between phytochemicals and DENV proteins, paving the way for designing novel NS3 and NS5 inhibitors from plant-derived compounds. While this study serves as a promising foundation, further experimental validation is essential to confirm the efficacy and safety of phytochemicals as potential NS3 and NS5 inhibitors for dengue treatment.

## 2. Results and Discussion

### 2.1. Virtual Screening and Molecular Docking

MD simulations, conformational and toxicity analyses, and logical screening made the drug discovery against viruses easier to identify possible inhibitors and comprehend the bio-physio-chemical basis of the inhibitor binding mechanisms [37]. To predict the bioactive compounds from plants and create medications that target the proteins that cause viral infection, molecular docking has been used extensively [38,39]. Usually, the docking process begins when a smaller molecule binds to a larger molecule, such as an enzyme or protein [40]. The current investigation started by selecting more than 17 plants that contained antiviral compounds. Nearly 898 antiviral medicinal phytochemicals from those plants were then shortlisted. The docking score was calculated using AutoDock Vina, and the binding propensity of the compounds against the dengue NS3 and NS5 proteins was computed and ranked. In this context of screening the compound library, a lower binding affinity was regarded as a good docking score. Each compound’s lowest affinity was chosen from a range of binding positions because this represents the lowest energy release during bond formation [41]. From the docking of the phytochemicals, their lower binding affinity compared to the inhibitor was examined. Based on ADME and toxicity profiles prediction and the lowest affinity, the top 15 compounds for NS3 protein, i.e., DIOSGENIN, (-)-Cinchonain IA, Cinchonain 1a, Stigmast-4-ene-3,6-dione; 23670-94-2, Ternatoside C, Dehydrocarpaine II, 3-Hydroxyglabrol, Sitostenone, silibinin, Rubiadin 1-methyl ether, Rubiadin, ellagic acid, Taraxerone, Taraxerol, Anthraquinone, Stigmasterol showing negative binding affinity (kcal/mol) of −8.0 to −9.4 and top the 15 compounds for NS5 protein, i.e., Bisandrographolide A, Taraxerol, Taraxerone, Cycloeucalenol, Friedelan-3-one, 24-Methylenecycloartanol, Dehydrocarpaine II, Carpaine, NSC 640467, beta-Amyrin, Diosgenin, oleanoic acid, Oleanolic Acid, Andrographidin A, and Methyl oleanolate showing negative binding affinity (kcal/mol) of −8.8 to −10.1 were selected (Supplementary Table S2). Additionally, the docking scores of both proteins were compared with known dengue inhibitors, as presented in Table 1.

Given that molecular interactions provide valuable insights into the molecular mechanisms within biological systems, we examined the interactions within the docked complexes. NS3 contains a trypsin-like serine protease called NS3pro, which consists of the N-terminal 180 amino acids [42] and has a catalytic triad consisting of His51, Asp75, and Ser135. Substituting the catalytic Ser135 with alanine rendered the NS3 protease enzymatically inactive [43]. The ultra-conserved residues in DENV2 NS3, shared with all serine proteases, such as the catalytic Ser135, along with Gly133, Gly136, Gly148, Leu149, and Gly153, are likely essential for preserving the enzyme’s 3D structure or shaping the substrate-binding pocket [44]. The NS3 cleavage activity showed a notable decrease due to the alanine substitutions at Val95 and Gln96 residues in the NS3 protease sequence [45]. The suggestion was made that these two residues are situated at the end of the NS2B binding cleft and play a role in the initial association of NS2B with NS3 and in the accurate processing at the NS2B/NS3 site [46]. The serine protease encoded by DENV is located within N-terminal 180 amino acid residues of the 618-residue multifunctional NS3 protein. Although this region tends to form aggregates and is enzymatically inactive, the central hydrophilic segment of the integral membrane protein NS2B (residues 49–95) can associate to form the active protease [47]. To pinpoint key determinants for substrate catalysis and binding within the active site of NS3 protease of DENV, residues like Leu115, Asp129, Gly133, Thr134, Tyr150, Gly151, Asn152, Ser163, and Ile165, located in the enzyme’s S1 and S2 pockets, were subjected to alanine substitution mutagenesis. The effects on enzyme activity were evaluated using fluorometric assays. Among these, Leu115, Asp129, Tyr150, and Ser163 were identified as lining the S1 subsite, while Asn152 was highlighted as a critical residue in the S2 subsite [48]. When the medicinal properties of a few chosen plants were examined, the majority of them demonstrated inhibitory activity against the dengue virus, suggesting possible dengue control. Dengue exhibited the strongest effects, but these plants also had antiviral, anti-inflammatory, anti-cancer, and antibacterial properties.

**Figure 1:**
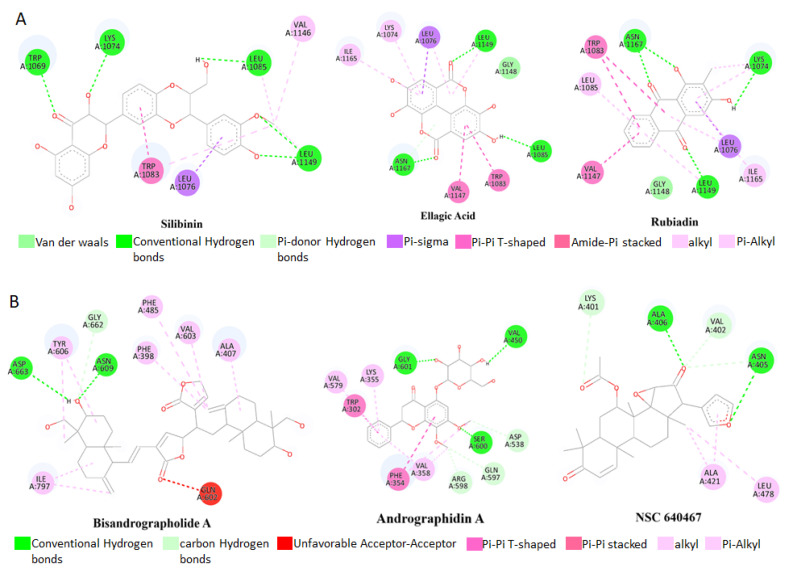
Molecular docking interaction with the targeted protein. The two-dimensional binding modes of selected lead compounds within the active sites of the complexes. (**A**); NS3 protein (PDB ID: 6MO0), (**B**); NS5 protein (PDB ID: 2J7W). Colors indicate interactions.

**Figure 2:**
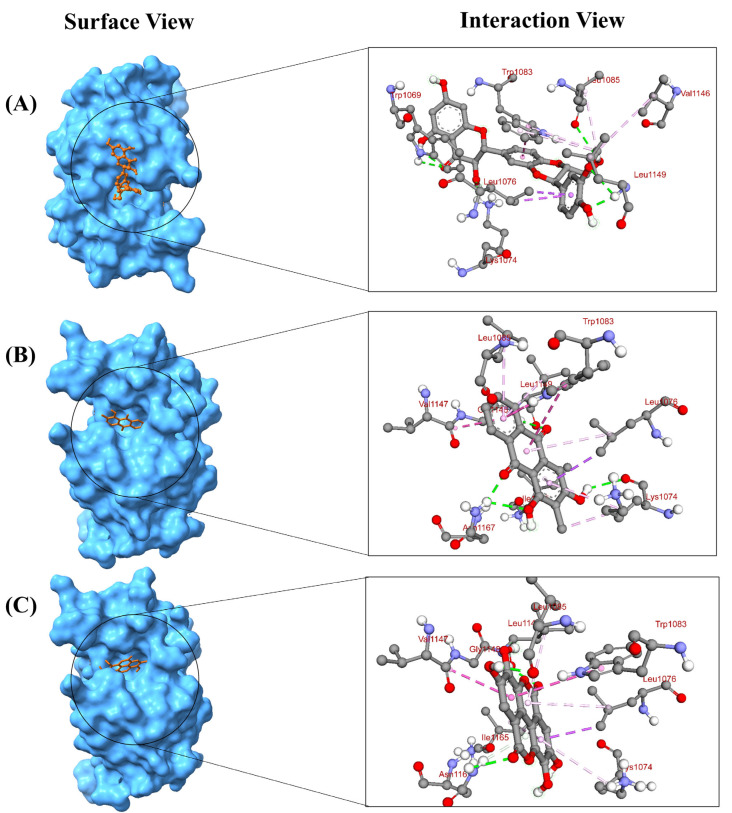
Molecular docking with the target NS3 protein and the different molecular orientations and interactions within the active sites. The three-dimensional (3D) binding modes with their surface view and interaction pose view of the selected lead compounds within the active sites of the complexes. (**A**) Silibinin, (**B**) Rubiadin, and (**C**) Ellagic Acid (PDB ID: 6MO0).

**Figure 3:**
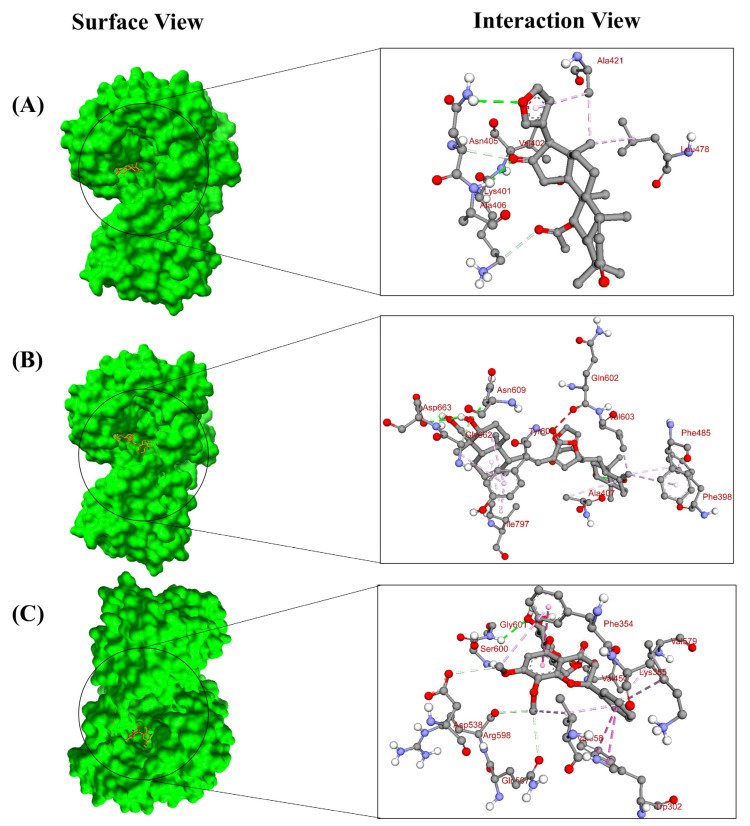
Molecular docking with the target NS5 protein and the different molecular orientations and interactions within the active sites. The three-dimensional (3D) binding modes with their surface view and interaction pose view of the selected lead compounds within the active sites of the complexes. (**A**) NSC 640467, (**B**) Bisandrographolide A, and (**C**) Andrographidin A (PDB ID: 2J7W).

Based on the lowest affinity, among the top 15 compounds, Silibinin, Rubiadin, and Ellagic Acid were identified as top potential candidates for the NS3 protein, and NSC 640467, Bisandrographolide A, and Andrographidin A were identified as top potential candidates for the NS5 protein (Figure 1). Their docking scores, predicted pIC50 scores, and non-bond interaction between proteins and compounds are summarized in Table 1. The top lead compounds demonstrated significant docking conformations, showing binding affinities exceeding −8.1 kcal/mol within the active site of the target proteins [21]. Results exhibited that Silibinin obtained a docking score of −8.5 kcal/mol and established four hydrogen bonds with amino acid residues Trp1069, Lys1074, Leu1149, and Leu1085. Moreover, it formed three hydrophobic bonds with residues Trp1083, Val1146, and Leu1076, thereby interacting with the NS3 protein. Conversely, Rubiadin demonstrated a docking score of −8.3 kcal/mol against NS3 and established three hydrogen bonds with Leu1149, Asn1167, and Lys1074 and five hydrophobic bonds with Leu1076, Trp1083, Val1147, Ile1165, and Leu1085 residues. Likewise, Ellagic acid exhibited a docking score of −8.2 kcal/mol against the target protein and showed three hydrogen bonds with Leu1149, Asn1167, and Leu1083. Furthermore, it formed five hydrophobic bonds with residues Leu1076, Trp1083, Val1147, Lys1074, and Ile1165. The docking results analysis of those lead compounds showed interaction with the target protein’s active sites and catalytic sites. These interactions suggest the lead compound’s capacity to break crucial active sites of the target protein. On the other hand, Bisandrographolide A interacts with the NS5 protein, demonstrating a docking score of −10.1 kcal/mol, establishes three hydrogen bonds with Asn609, Asp663, and Val603, and forms eight hydrophobic bonds. NSC 640467 exhibited a docking score of −9.3 kcal/mol and formed six hydrogen bonds with residues Asn405, Ala406, Lys401, Val402, Asn405, and Ala407, and additionally established three hydrophobic bonds against the target protein. In contrast, Andrographidin A achieved a docking score of −8.9 kcal/mol and established six hydrogen bonds with Ser600, Gly601, Val450, Asp538, Gln597, and Arg598 and five hydrophobic bonds, thereby interacting with the NS5 protein. Docking results showed that all lead compounds indicate their capacity to disrupt the protein’s active sites. The 2D chemical structure of the compounds for NS3 and NS5 of the MD interaction is demonstrated in the Supplementary Figure S1. The three-dimensional (3D) surface view and pose view of the potential compounds of the targeted NS3 and NS5 proteins are demonstrated in Figure 2 and Figure 3.

### 2.2. Re-Screening Through ML

**Figure 4:**
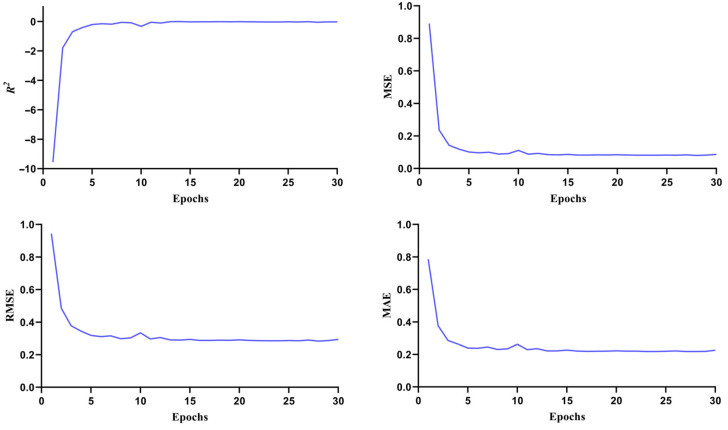
The accuracy plots of the Dengue inhibitors regression model illustrate various statistical parameters of the machine learning model.

The machine learning approach utilizes various artificial neural network frameworks to analyze datasets and predict molecular properties and bioactivities. By leveraging chemical, topological, and biological data patterns, it processes large compound libraries to identify potential drug candidates [49,50]. The screening was conducted to identify compounds with inhibitory activity against the target protein, specifically those targeting Dengue virus type 2, which were then used to develop multiple IC50-based regression models. The performance and quality of the selected model were assessed using evaluation metrics such as the coefficient of determination (R^2^), mean square error (MSE), root mean square error (RMSE), and mean absolute error (MAE) [37]. These parameters indicate how well the regression model fits the data and the differences between the predicted and actual values. IC50 is a vital metric in drug-discovery research. It demonstrated the concentration of compounds essential to inhibit a biological function by 50%, which strengthen the prediction accuracy, reducing experimental costs and accelerating drug development [51]. We built a regression model using the dengue inhibitors’ IC50 data (845 compounds) with a default PubChem fingerprint and standard neural network parameters, achieving good performance with an R^2^ of 0.68, where the predicted pIC50 scores closely matched the experimental data, demonstrating the high accuracy of the developed deep learning model for dengue inhibitors (Figure 4). The high R^2^ indicates the model’s well-fitted and good performance, while the low MSE, RMSE, and MAE values signified its high accuracy. The selected compounds were tested in the regression model against biologically active inhibitors for the NS3 and NS5 proteins of the Dengue virus. For NS3, Silibinin, Rubiadin, and Ellagic Acid showed pIC50 values of 4.76, 4.70, and 4.35, respectively, while NS5, NSC 640467, Bisandrographolide A, and Andrographidin A had pIC50 values of 4.43, 4.71, and 4.84, respectively, indicating that these compounds were biologically active and capable of inhibiting the proteins.

### 2.3. ADME/Toxicity Analysis

Factors such as physicochemical properties, absorption, distribution, toxicity, selectivity, pharmacokinetics, and mechanism of action play a crucial role in evaluating a compound’s potential as a drug candidate. Some of these factors of the six leading candidates identified through the ADMET are provided in Table 2. Drug-likeness property of a compound makes a phytochemical more likely to succeed as a drug candidate [52]. This is tightly linked to phytochemicals’ physicochemical properties, which determine how they interact with biological systems and behave within the body [53]. ADMET, drug-likeness, and bioactivity predictions were performed for six compounds analyzed. The selected phytochemicals met Lipinski’s ‘Rule of Five’, suggesting they qualify as potential lead compounds. Among them, Rubiadin, Ellagic Acid, and NSC 640467 showed superior gastrointestinal absorption, and NSC 640467, Bisandrographolide A, and Andrographidin A were recognized as substrates for p-glycoprotein. All of the compounds except Rubiadin showed an inability to cross the blood-brain barrier. Additionally, Bisandrographolide A exhibited the highest score for synthetic accessibility compared to the other compounds.

In toxicity parameters, Rubiadin, Ellagic Acid, and Andrographidine A showed prominent activity in mutagenicity, carcinogenicity, and cytotoxicity, respectively, while showing inactivity for hepatotoxicity. Alternatively, Silibinin, NSC 640467, and Bisandrographolide A showed inactivity in all the previously mentioned parameters. All of the six compounds were classified as low toxic and assigned to toxicity class from 4 to 5. The ADMET analysis confirmed that all six ligands complied with Lipinski’s rules, a key criterion for identifying potential lead compounds. Additionally, pharmacokinetics and toxicity assessments showed that the selected lead compounds demonstrated satisfactory results, as presented in Table 2 and Table 3.

### 2.4. Molecular Dynamics Simulation

**Figure 5:**
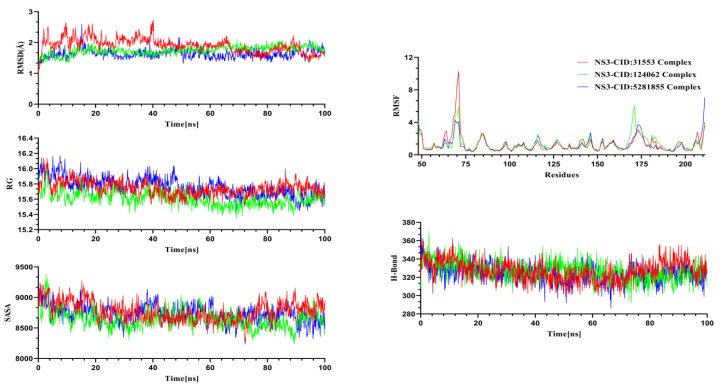
Molecular dynamics simulation trajectories were analyzed for the selected ligand–protein complexes involving the NS3 protein. CID:31553; Silibinin, CID:124062; Rubiadin, CID:5281855; Ellagic acid.

**Figure 6:**
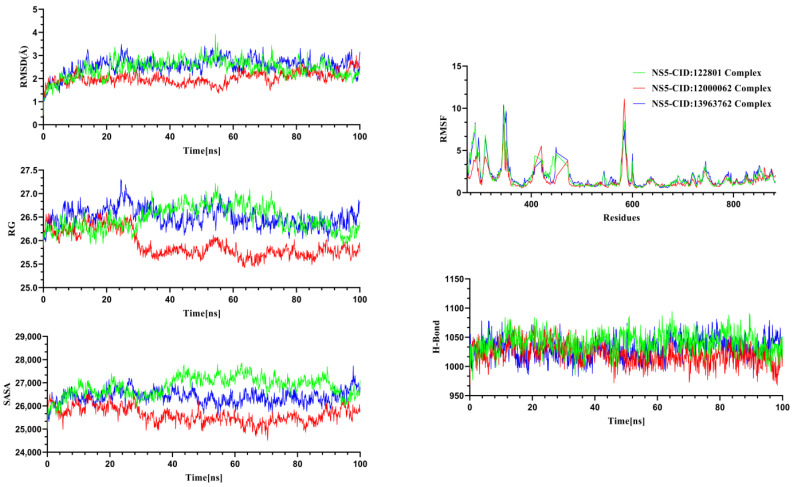
Molecular dynamics simulation trajectories were analyzed for the selected ligand–protein complexes involving the NS5 protein. CID:12000062; Bisandrographolide A, CID:122801; NSC 640467, CID:13963762; Andrographidin A.

Molecular dynamics simulations were performed for the NS3 and NS5 proteins in complex with their respective ligands to investigate structural changes under dynamic conditions. Insights gained from the MD trajectories were utilized to assess the structural stability of the protein–ligand complexes. Key parameters, including RMSD, hydrogen bonding, RMSF, SASA, and RG, were analyzed from the simulation data [54]. RMSD analysis was conducted for the alpha carbon atoms, RMSF was used to evaluate the flexibility of amino acid residues, RG assessed the rigidity and compactness of the protein, and SASA was analyzed to determine protein volume and expansion [37]. The MD simulation for the system was performed for 100 ns to gain insights into structural behavior, binding interactions, and flexibility of the Silibinin complex, Rubiadin complex, and Ellagic Acid complex (Figure 5) for the NS3 protease and NSC 640467 complex, Bisandrographolide A complex, and Andrographidin A complex for the NS5 protease (Figure 6).

The graph for NS3 (Figure 5) demonstrated that at the start of the simulations, Silibinin, Rubiadin, and Ellagic Acid have a lower trend in RMSD, indicating greater structural stability over the simulation period. The complexes reached a stable state after 40 ns and maintained their integrity through to the final frames of the simulations. Silibinin exhibited a higher RMSD trend and more fluctuations between 5–45 ns, suggesting a greater flexibility in the complex. However, the complexes ultimately stabilized. Throughout the simulations, the RMSD remained below 2.7 Å for all complexes, indicating overall stability. The RMSF analysis revealed that most residues had an RMSF below 2.5 Å, further confirming the stability of the complexes. Additionally, the SASA of the simulated complexes was analyzed, where larger SASA values indicated an expansion of the protein surface area, and lower SASA values suggested a more compact structure of the complexes. The graph demonstrated that Rubiadin exhibited a smaller SASA trend and fewer fluctuations compared to the other complexes, suggesting that when the ligand binds to the protein, the surface area does not expand, leading to increased stability. The radius of gyration (RG) from the simulation trajectories indicated that a larger RG corresponds to a more mobile complex, while a smaller RG reflects a more rigid structure. From 50 ns onward, the complexes displayed lower RG values, indicating they became more compact and structurally stable. The other complexes showed minimal divergence in their trajectories. Hydrogen bonding played a key role in determining the stable state of the complexes, with the graph showing that all complexes exhibited minimal hydrogen bond patterning variations [55].

The graph for NS5 (Figure 6) demonstrated the simulations of NSC 640467, Bisandrographolide A, and Andrographidin A complexes. The RMSD trajectories of the complexes exhibited variations, with values peaking at 3.9 Å, where the NSC 640467 complex exhibited an initial rise followed by a period of relatively steady fluctuations from 20 to 54 ns of 2.2 Å to 3.9 Å, followed by a downward trend in its path. The Bisandrographolide A complex initially displayed stability, exhibiting downward trend fluctuations between 38 and 59 ns. The Andrographidin A complex had maintained the integrity until the simulation final images. The RMSD trajectories for all of these complexes suggest that the compounds maintained stable configurations during the simulation, with no significant structural deviations from their initial states, indicating that the complexes were overall stable. The RMSF of the complexes showed that maximum residues had an RMSF of less than 2.6 Å. This indicated that the complexes exhibited flexibility, highlighting the proteins’ overall stable structural framework. Furthermore, the SASA of the simulated complexes showed the extent of a molecule’s surface area that is accessible to solvent molecules [56]. The graph demonstrated that Bisandrographolide A had a smaller SASA trend and a lower degree of departures compared to other complexes, indicating that when the ligand binds to the protein, surface area does not expand and the stability increases. Throughout the simulation, the NSC 640467 complex exhibited fluctuating trajectories, indicating a considerable level of flexibility and a substantial increase in the surface area expansion of the protein. In contrast, the Andrographidin A complex displayed more moderate expansions in the protein surface area. The RG from the simulated trajectories, with larger RG indicating a more mobile character of the complexes and the lower RG indicating a stiffer structure. The RG trajectories of the NSC 640467, Bisandrographolide A, and Andrographidin A complexes displayed similar trends up to 24 ns. On the other hand, the Bisandrographolide A complex exhibited lower fluctuation compared to the other complexes throughout the simulations. The Bisandrographolide A exhibited its lowest RG value at 63 ns of 25.44 Å, while NSC 640467 and Andrographidin A exhibited 25.92 Å and 25.98 Å at 16 ns and 90 ns, respectively. The Bisandrographolide A complex noticeably exhibited more variation compared to the other complexes during the simulation. However, its behavior later aligned with the patterns seen in the other three complexes, eventually stabilizing over time. The structural stability and the folding characteristics of the protein-ligand interactions remained steady throughout the simulation. In determining the stable state of complexes, the hydrogen bond plays a crucial role. The graph shows that all complexes showed lower degrees of the hydrogen bond patterning aberrations [55]. The hydrogen bond trajectories of the three complexes stayed consistent throughout the simulation, reflecting the stability and low fluctuation of each complex.

In summary, this study aimed to identify novel potential phytochemical-based inhibitors—Silibinin, Rubiadin, Ellagic Acid, NSC 640467, Bisandrographolide A, and Andrographidine A—targeting NS3 and NS5 through molecular docking and molecular dynamics simulations. Those identified compounds have potential activities against the Dengue virus. Ellagic acid exhibits potential antiviral activity against the DENV. Ellagic acid, derived from *Punica granatum* (pomegranate), inhibits the NS2B–NS3 protease across all DENV serotypes. Also, it is identified that ellagic acid efficiently interacts with DENV envelope proteins, indicating its potential to hinder viral entry into host cells [57,58]. Interestingly, ellagic acid had been reported to exhibit antiviral activity against the Zika virus and human rhinoviruses [59]. Rubiadin is a powerful molecule with anticancer, antiosteoporotic, hepatoprotective, neuroprotective, anti-inflammatory, antidiabetic, antioxidant, antibacterial, antimalarial, antifungal, and antiviral properties [60]. Bisandrographolide has also been shown to exhibit antiviral activity against a variety of viruses, including influenza A and B viruses, respiratory syncytial virus (RSV), and dengue virus [61,62]. Based on the comprehensive analysis, six lead compounds appear to be promising candidates for treating Dengue. However, further laboratory experiments are essential to confirm their anti-dengue properties, potentially providing new alternatives for Dengue treatment.

## 3. Materials and Methods

### 3.1. Retrieval and Preparation of Proteins

The DENV NS3 (GenBank Accession ID: P07564) and NS5 (GenBank Accession ID: AFN80338) protein’s three-dimensional coordinate structures were obtained from the RCSB Protein Data Bank in the format of pdb [63]. Retrieve the PDB ID: 6MO0 for NS3 and PDB ID: 2J7W for NS5. Then, the protein structures were prepared by eliminating all heteroatoms and water molecules using PyMol v3.1 and the Discovery Studio client v24.1. GROMOS96 43B1 force was used for energy minimization and optimization of clean proteins using Swiss-PDB Viewer [37].

### 3.2. Preparation of Ligand

Initially, 17 medicinal plants and their corresponding 898 phytochemicals identified by GC-MS results and exhibiting were collected through a rigorous literature review that selected based on their antiviral properties and reported bioactivity against viral infections in the literature (Supplementary Table S1) [56]. The structures of all phytochemicals were downloaded from the PubChem Database in 3D sdf format for virtual screening [64]. Prior to performing molecular docking studies, the ligand’s energy was minimized using PyRx software v0.8 with the MMFF94 force field and 2000 steps of the steepest descent optimization algorithm [37].

### 3.3. Structure-Based Virtual Screening

Computer-generated 3-D structure of ligands is placed into a receptor structure using molecular docking in a range of orientations, conformations, and locations. In this study, a molecular docking study was carried out using the PyRx V1.1 virtual screening software to identify the precise residues in the target protein that bind with the different ligands [37]. The energy-minimized protein (in pdb format) was loaded in PyRx and made into a macromolecule. Subsequently, the phytochemical libraries were opened in PyRx. All the energy-minimized ligands (in pdbqt) were selected, and the AutoDock Vina v4.2.6 was used for docking these ligands with target proteins. A grid box [65] was generated, and the size of the grid box (in Angstrom) was maximized for NS3, (X = 44.2946, Y = 48.5186, Z = 42.0140) and NS5, (X = 70.5738, Y = 71.2430, Z = 70.4744) so that all the ligands could appear inside the box and then docking was started. The binding affinity of the ligands appeared as negative values by kcal/mol as a unit. The binding affinities of all the ligands were recorded in CSV files, and those who had the highest values of binding affinities (−8.0 kcal/mol) were prioritized [66,67]. The top 50 ligands were selected according to their best binding affinities. The re-docking process was carried out in the same way for NS3 and NS5 proteins. Re-docking of known inhibitors that were bound with protein structures to compare binding affinities with our screening phytochemicals. Finally, select 15 ligands based on their highest negative binding affinities of both proteins for further re-screening through machine learning approaches. The binding affinity and the best binding position were visualized in the Py-Mol viewer [68]. Recent studies showed that the identification of potential inhibitors using the docking study against NS3 and NS5 was carried out by several groups [54,69,70].

### 3.4. Re-Screening Through Machine Learning (ML)

Phytochemicals with a docking score greater than −8.0 kcal/mol were selected for further evaluation using a deep learning approach. Recurrent Neural Networks (RNN) were employed to develop predictive models based on the CHEMBL5980 dataset (Supplementary Table S2), which contains inhibitors targeting the Dengue virus type 2. The Deep Screening server was utilized for this process, providing an average area under the ROC curve (AUC) of 0.86 and a median AUC of 0.89 across multiple deep-learning models [71]. These results indicate strong performance in identifying potential candidates. The dataset was processed for fingerprint generation using the PubChem fingerprint method, producing 881 unique fingerprints with the help of PaDEL software v2.21 [72]. The hyperparameters for the developed model were manually configured, including a learning rate of 0.0001, three hidden layers, and neurons set at 2000, 700, and 200 for each layer, respectively. The hidden layers utilized an activation function, while the output layer employed a sigmoid function. The predicted pIC50 score was then calculated, and this model was used to re-screen the top 15 docked compounds for both proteins, with the results presented in the Supplementary Table S3. Predicted pIC50 was mathematically determined using this following equation:

pIC50 = −log_10_IC50 (M) = 9 − log_10_IC50 (nM)

The DeepScreening platform for deep learning-based virtual screening operates through a straightforward framework: (i) Dataset Preparation—Choose a specific target to train the deep neural network (DNN). (ii) Feature Selection—Determine the molecular features required for vectorization. (iii) Model Parameters—Define key parameters for training regression models. (iv) Virtual Screening—Conduct virtual screening against a chemical library. DeepScreening is an advanced, fully automated screening server that seamlessly integrates multiple technologies and tools for data preprocessing, model development, and virtual screening [71].

### 3.5. ADME/Tox Analysis of Phytochemicals

Absorption, distribution, metabolism, excretion, and toxicity (ADMET) of all of the compounds were calculated using ADMETlab 3.0 and pKCSM. The canonical smiles of the ligands were collected from the PubChem database, and those smiles were used to find out the pharmacokinetics of the ligands. To determine the pharmacokinetic properties and drug-likeness, the Lipinski’s Rule of Five (LRF) was applied where the rule stands for four factors as lipophilicity, hydrogen donors, hydrogen acceptors, and the molecular weight of the phytochemicals [68]. The toxicity of the ligands was determined using ProTox-3.0. This software’s toxicity level ranges from 1 to 6, where 1 refers to the most toxic and 6 to nontoxic. After all the analysis, the best three ligands were selected for each protein.

### 3.6. Computational Dynamics Study

Molecular docking is one of the most popular techniques for identifying a ligand’s primary binding position within a protein binding site [73]. In a synthetic setting, molecular dynamics (MD) simulation aids in identifying and verifying the entropic effects and structural flexibility of protein–ligand complexes. To eliminate unwanted contacts between solute and solvent water, energy minimization and equilibration simulations were carried out before the production simulations [37]. The complex was heated, equilibrated, and minimized in each simulation. The solute and lipid chains were subjected to a harmonic restraint of 10 kcal mol^−1^ Å^−2^ in the first step of energy minimization, and thereafter all atoms were free to move [74]. The predictions from the docking investigation were verified using MD simulation using YASARA software v25.1.13 [37]. The force field AMBER14 [75] was applied for this simulation. The structural integrity was compared to the complexes using the dengue NS3 and NS5 proteins as a control method [37]. Long-range electrostatic interactions were calculated using the particle mesh Ewald method, whereas short-range van der Waals and Coulomb interactions were examined using an 8 Å cutoff radius. The system’s overall environmental parameters were 298 K, pH 7.4, and 0.9% NaCl. The system was minimized using the steepest descent algorithm. The temperature and pressure were controlled using a Langevin thermostat and a Monte Carlo barostat, respectively. Using a time step of 1.25 fs, the MD simulation trajectories were stored every 100 ps. For every complex, several parallel paths were run to guarantee simulation convergence [37]. The root means square deviation (RMSD), the root means square fluctuation (RMSF), the radius of gyration (RG), the solvent accessible surface area (SASA), and the number of hydrogen bonds were all analyzed throughout the 100 ns MD simulation exercise [76,77,78,79]. GraphPad Prism v10.3 was used to visualize the MD graphs.

## 4. Conclusions

The DENV NS proteins play diverse and vital roles in ensuring the survival of the virus, which opens up possibilities to exercise many researches to find the best antiviral target. Inhibitors of NS proteins should be of great benefit in combating infections by viruses. With the current urgency in finding the best treatment for dengue, this study identified potential inhibitors as anti-dengue agents targeting NS proteins which is a specifically interesting molecular target for antiviral compounds because of its central role in the viral life cycle. Six compounds, selected based on molecular docking and inhibitory pIC50 scores—Silibinin, Rubiadin, Ellagic Acid, NSC 640467, Bisandrographolide A, and Andrographidin A—having activities against the DENV were further evaluated for their other properties. ADME and toxicity analysis confirmed their pharmacokinetic properties and safety, while MD simulations validated their structural stability at the target protein’s active site, highlighting their potential as inhibitors and potential usefulness in drug discovery of DENV. However, some time compounds of docking experiments are not in line with the in vitro and in vivo experiments, which are the potential limitations of the computational work. Therefore, further in vitro and in vivo studies are necessary to validate the efficacy and safety of the compounds, which we could not perform in this experiment. Future work will focus on enzymatic inhibition assays, cellular cytotoxicity tests, and animal models to confirm antiviral potential against Dengue.

## Figures and Tables

**Table 1:** Docking scores, predicted inhibitory potency (pIC50) score, and non-bond interaction between proteins and six selected lead compounds compared with dengue known inhibitor Balapiravir.

Compound Name and CID	Binding Affinity (kcal/mol)	Predicted pIC50 Score	Residue in Contact	Interaction Type	Bond Distance in Å
Silibinin (31553)	−8.5	4.76	TRP1069	H Bond	2.72728
			LYS1074	H Bond	2.15489
			LEU1149	H Bond	2.91918
			LEU1085	H Bond	2.95107
			TRP1083	hydrophobic	4.31848
			VAL1146	hydrophobic	5.21579
			LEU1076	hydrophobic	4.46035
Rubiadin (124062)	−8.3	4.7	LEU1149	H Bond	2.51707
			ASN1167	H Bond	2.13321
			LYS1074	H Bond	2.54066
			LEU1076	hydrophobic	2.86341
			TRP1083	hydrophobic	5.94837
			VAL1147	hydrophobic	3.92637
			ILE1165	hydrophobic	5.16293
			LEU1085	hydrophobic	4.94692
Ellagic acid (5281855)	−8.2	4.35	LEU1149	H Bond	2.23803
			ASN1167	H Bond	2.53243
			LEU1085	H Bond	2.04294
			LEU1076	hydrophobic	2.66139
			TRP1083	hydrophobic	5.50333
			VAL1147	hydrophobic	4.24805
			LYS1074	hydrophobic	4.87666
			ILE1165	hydrophobic	5.22632
Balapiravir (11691726)	−6.6	4.11	GLY1087	H Bond	2.03115
			VAL1146	H Bond	2.21827
			LYS1074	H Bond	2.81251
			LEU1149	H Bond	2.76025
			ASN1167	hydrophobic	2.07438
			GLY1148	hydrophobic	4.41981
			LEU1176	hydrophobic	4.12744
			ILE1165	hydrophobic	4.10504
Bisandrographolide A (12000062)	−10.1	4.43	ASN609	H Bond	2.23313
			ASP663	H Bond	3.03945
			VAL603	H Bond	2.51134
			ALA407	hydrophobic	5.0847
			ILE797	hydrophobic	4.3585
			VAL603	hydrophobic	4.12083
			ILE797	hydrophobic	4.55821
			PHE398	hydrophobic	5.22525
			PHE485	hydrophobic	4.97929
			TYR606	hydrophobic	5.23019
			TYR606	hydrophobic	5.2332
NSC 640467 (122801)	−9.3	4.71	ASN405	H Bond	2.89725
			ALA406	H Bond	2.31095
			LYS401	H Bond	2.64717
			VAL402	H Bond	2.27594
			ASN405	H Bond	2.26522
			ALA407	H Bond	3.05968
			ALA421	hydrophobic	3.65796
			LEU478	hydrophobic	4.98358
			ALA421	hydrophobic	4.94432
Andrographidin A (13963762)	−8.9	4.84	SER600	H Bond	2.3857
			GLY601	H Bond	2.66425
			VAL450	H Bond	2.78778
			ASP538	H Bond	2.61398
			GLN597	H Bond	2.73499
			ARG598	H Bond	2.43382
			TRP302	hydrophobic	4.19854
			PHE354	hydrophobic	5.25107
			VAL358	hydrophobic	4.62125
			LYS355	hydrophobic	5.30999
			VAL579	hydrophobic	4.86855
Balapiravir (11691726)	−6.9	4.11	GLN597	H Bond	2.87859
			ARG598	H Bond	2.99243
			SER600	H Bond	3.02471
			GLN602	H Bond	2.32165
			GLY599	H Bond	2.86808
			LYS575	hydrophobic	4.67156
			VAL577	hydrophobic	4.42977
			VAL579	hydrophobic	3.64284

H indicates hydrogen.

**Table 2:** Physicochemical and pharmacological characteristics of the six leading candidates identified through the ADMETlab 3.0 web servers.

	Properties	Silibinin	Rubiadin	Ellagic Acid	NSC 640467	Bisandrographolide A	Andrographidin A
Physicochemical Properties	MW (g/mol)	482.441	254.24	302.19	466.57	664.88	462.45
	Heavy Atoms	35	19	22	34	48	33
	Aromatic Atoms	18	12	16	5	0	12
	Rotatable bonds	4	0	0	2	8	6
	H-Bond acceptors	10	4	8	6	8	10
	H-Bond donors	5	2	4	0	4	4
	TPSA (Å^2^)	155.14 Å^2^	74.60 Å^2^	141.34 Å^2^	86.11Å^2^	133.52 Å^2^	144.14 Å^2^
Lipophilicity	Log P_o/w_ (Cons)	2.36	2.18	1.31	4.62	5.36	0.58
Water Solubility	Log S (ESOL)	−4.14	−3.82	−2.94	−5.31	−7.26	−3.01
Pharmacokinetics	GI Absorption	low	high	high	high	low	low
	BBB permeant	no	yes	no	no	no	no
	P-GP Substrate	no	no	no	yes	yes	yes
Drug likeness	Lipinski	yes	yes	yes	yes	yes	yes
Medi. Chemistry	Synth. accessibility	4.92	2.52	3.17	6.34	8.00	5.33

**Table 3:** Toxicity assessment of the six lead compounds through ProTox-3.0 web server.

Target	Silibinin	Rubiadin	Ellagic Acid	NSC 640467	Bisandrographolide A	Andrographidine A
Hepatotoxicity	inactive	inactive	inactive	inactive	inactive	inactive
Carcinogenicity	inactive	inactive	active	inactive	inactive	inactive
Mutagenicity	inactive	active	inactive	inactive	inactive	inactive
Cytotoxicity	inactive	inactive	inactive	inactive	inactive	active
LD_50_ (mg kg^−1^)	2000	7000	2991	555	452	3000
Immunotoxicity	active	active	inactive	active	active	active
Toxicity Class	4	5	4	4	4	5

## Data Availability

The data supporting this study’s findings are within the article and Supplementary File.

## Supplementary Materials

The following supporting information can be downloaded at: https://www.mdpi.com/article/10.3390/ph18040566/s1, Table S1; Library of a complete list of phytochemicals with their CID obtained from 17 medicinal plants in this study with references [80,81,82,83,84,85,86,87,88,89,90,91,92,93,94,95,96,97,98,99,100,101,102,103,104,105,106,107,108,109,110,111,112,113,114,115,116,117,118,119,120,121,122,123,124,125,126]. Table S2; Recurrent Neural Networks (RNN) was employed to develop predictive models based on the CHEMBL5980 dataset, which contains inhibitors targeting the Dengue virus type 2. Table S3; Binding Affinity (kcal/mol) and Predicted pIC50 scores of the phytochemicals for NS3 and NS5 protein, as predicted by AutoDock Vina and Machine learning approach, respectively. Figure S1; The 2D chemical structures of (A) Silibinin, Rubiadin, and Ellagic Acid, and (B) NSC 640467, Bisandrographolide A, and Andrographidin.
