# Supplementary material for: Identification of Potential Inhibitors Targeting Non-Structural Proteins NS3 and NS5 of Dengue Virus Using Docking and Deep Learning Approaches
Source: Pharmaceuticals (Basel). 2025 Apr 13;18(4):566. doi: 10.3390/ph18040566 (PMC12030398; doi:10.3390/ph18040566)
Supplement: Supplementary file 1 [file pharmaceuticals-18-00566-s001.zip › Table S1.pdf]

Supplementary Table S1. Library of a complete list of phytochemicals with their CID obtained from 17 medicinal plants in this study.

Plant Name	Phytochemical name	Pubchem CID	Reference
1. **Andrographis paniculata**	Dihydrocytisine	91747228	[80]
	Ammodendrine	442625	
	Spartein	644020	
	Phytate	890	
	Hydroxylupanine	73404	
	Kaempferol	5280863	
	Sapogenin	101810	
	Ribalinidine	336322	
	Anthocyanin	29231	
	Flavone	10680	
	Aphyllidine	12306738	
	Proanthocyanidin	108065	
	Isolupanine	15939852	
	Narigenin	439246	
	1,1,3-triethoxy-Propane	24624	[81]
	Tetradecanoic acid	11005	
	n-Hexadecanoic acid (Palmitic acid)	985	
	9,12-Octadecadienoyl chloride	98987	
	9,12-Octadecadienoic acid (Linoleic acid)	3931	
	9,12,15-Octadecatrienoic acid (Alpha-linolenic acid)	5280934	
	1,2-benzenedicarboxylic acid (Phthalic acid)	1017	
	diisooctyl ester	33934	
	p-Hydroxybenzoic acid methyl ester	7456	
	Androstan-17-one	10880319	
	Squalene	638072	
	14-deoxy-15-isopropylidene-11	637300	[82]

	12-didehydroandrographolide	5708351	
	Andrographolactone	44206466	
	Neoandrographolide	9848024	
	14-deoxy 11	5708351	
	Andrographolide	5318517	
	Deoxyandrographolide	21679042	[83]
	Methyl Caffeate	689075	
	Quercetin	5280343	
	Methyl vanillate	19844	
	Vanillic acid	8468	
	p-Hydroxybenzoate	54675830	
	Caffeic acid	689043	
	Onysilin	12041831	
	Cinnamic acid	444539	
	Ferulic acid	445858	
	Adipic acid	196	
	Skullcapflavone	13889022	
	2-Propenamide,N-[2-(dimethylamino)ethyl]	70218	[84]
	1-Benzylamino-2-benzyloxyethane	562448	
	3-Selenetanol,3-(4-methoxyphenyl)	591602	
	1,3-Dioxolane,2-(3-methoxypropyl)-2-methyl	560002	
	7-Tridecanol	136715	

	2-Iodo-cinnamic acid	5372934	
	3-Nitrobenzyl iodide	77567	
	Sucrose	5988	
	Cellulose	16211032	
	3,7,11,15-Tetramethyl-2-hexadecen-1-ol (Phytol)	5280435	
	2-Pentadecanone 6,10,14-trimethyl	10408	
	Phthalic acid, hex-3-yl isobutyl ester	91719722	
	Di-sec-butyl Phthalate	249496	
	Tetradecanoic acid, 10,13-dimethyl-, methyl ester	554145	
	l-(+)-Ascorbic acid 2,6-dihexadecanoate	54722209	
	Phthalic acid, hept-4-yl isobutyl ester	91720280	
	Eicosane	8222	
	1-Hexyl-2-nitrocyclohexane	544017	
	2-Nonadecanol	98188	
	6-Tetradecanesulfonic acid, butyl ester	551402	
	9-Hexacosene	5363630	
	Sulfurous acid, cyclohexylmethyl isobutyl ester	6421696	
	Sulfurous acid, butyl nonyl ester	6420801	
	14-deoxy-11-oxoandrographolide	9975052	[85]
	14-deoxyandrographolide	11624161	

	Panicolin	5320399	
	5,7,2’,3’-tetramethoxyflavanone	21579286	
	5-hydroxy-7,2’,3’-trimethoxyflavone	12135219	
	3-O-beta-D-Glucopyranosyl-14,19-dideoxyandrographolide	11576609	[86]
	Luteolin	5280445	
	Andrographidine C	5318484	
	Andrograpanin	11666871	
	Bisandrographolide	12000062	
	Andrographidin A	13963762	
	14-Deoxy-12-Hydroxyandrographolide	38350572	
	8,17-Epoxy-14-Deoxyandrographolide	44575263	
	Andrographiside	44593583	
	Isoandrographolide	101243415	
	6-gingerol	442793	
	3-hydroxyflavone	11349	
	cyanidin	128861	
	Octadecadiynoic acid	23615513	[87]
	Stigmast-5-en-3-ol	22012	
	Phenanthrenecarboxylic acid	10569	
	Phytosterols	12303662	

	13,15-octacosadiyne	578154	
2. **Alternanthera philoxeroides**	2,5-bis(1,1-dimethylethyl)-	2374	[88]
	2-(2-methoxycarbonylamino-5-nitrophenylthio)-	548128	
	1,4-Benzenediol	785	
	3-Methyl-4-(phenylthio)-2-prop-2-enyl-2,5- dihydrothiophene 1,1-dioxide	6420230	
	4-Pyridinecarboxamide, 6-bromo-4,5- dicyano-1,2,3,4-tetrahydro-3,3-dimethyl-2- [[(1-methylethylidene)amino]oxy	5298841	
	L-Cysteine, N-(trifluoroacetyl)-, butyl ester, trifluoroacetate (ester)	91691250	
	5-Nitro-1,3-benzothiazol-2-amine	2754759	
	Isoshyobunone	5318673	
	Bromperidol	2448	
	Fucoxanthin	5281239	
	2-Chloro-5-(1,2,4-triazol-4-yl)aniline	28307713	
	Thiamphenicol	27200	
	Kinetin	3830	
	1-(2-Nitrophenyl)piperazine	100949	
	3-Chloro-4-(1,2,4-triazol-4-yl)benzoic acid	42647323	
	3-Bromo-N-(2-thiazolyl) benzamide	603119	
	Isocalamendiol	12302240	
	5 Cyclopentaneundecanoic acid, methyl ester	535041	

	Dihydrosteviobiside	579254	
	Lysergic Acid	6717	
	Thiocolchicine	17648	
	4-Bromo-5-nitro-1H-imidazole	135408628	
	10-Octadecenal	545646	
	Aristolochic acid	2236	
	Chalcone	637760	
	N-trans-Feruloyl-3-methyldopamine	5352115	[89]
	N-trans-Feruloyl-tyramine	5280537	
	N-cis-Feruloyl-tyramine	6440659	
	Indole-3-carboxaldehyde	10256	
	Indole-3-carboxylic acid	69867	
	Pheophytin A	135398712	
	2-Hydroxy-3-methylanthraquinone	10889963	
	Rubiadin	124062	
	Rubiadin-1-methyl ether	96191	
	Alternanthin	44258156	
	Kaempferol	5280863	
	Luteolin 8-C-E-propenoic acid	16109839	
	Luteolin	5280445	

	Quercetin	5280343	
	Rutin	5280805	
	Demethyltorosaflavone D	44258148	
	Blumenol A	5280462	
	4,5-Dihydroblumenol A	21630916	
	Chlorogenic acid	1794427	
	p-Coumaric acid	637542	
	Ferulic acid	445858	
	p-Hydroxybenzoic acid	135	
	Vanillic acid	8468	
	Salicylic acid	338	
	Syringic acid	10742	
	Chikusetsusaponin IVa	13909684	
	Chikusetsusaponin IVa methyl ester	637855	
	3β-Hydroxystigmast-5-en-7-one	14779505	
	stigmasta-5, 22-dien-3-ol	5280794	
	Cycloeucalenol	101690	
	24-Methylenecycloartanol	94204	
	Oleanoic acid or oleanolic acid	485707	
	Phytol	5280435	

	Ursolic acid	64945	
	Azelaic acid	2266	
	Ascorbic acid	54670067	
3. **Carica papaya**	Papain	5249653	[90]
	Caffeic acid	689043	
	Myricetin	5281672	
	Rutin	5280805	
	Quercetin	5280343	
	Benzyl isothiocyanate	2346	
	Kaempferol	5280863	
	5-Deoxykaempferol	5281611	[91]
	Ascorbic acid	54670067	
	Carpaine	442630	
	Dicoumarol	54676038	
	Coumarin	323	
	Folic acid	135398658	
	Cystine	67678	
	Homocysteine	91552	
	Phenylalanine	6140	
	p- Coumaryl alcohol	5280535	

	Umbelliferone	5281426`	
	Caffeoyl alcohol	5282096	
	Methyl nonyl ketone	8163	
	Neochlorogenic acid	5280633	[92]
	Apigenin	5280443	
	p-Hydroxybenzoic acid	135	
	Salicylic acid	338	
	Hyperoside	5281643	
	Dehydrocarpaine I	131750991	
	Dehydrocarpaine II	131750992	
	Chrysin	5281607	
	Gentisyl alcohol	188287	
	Benzyl isothiocyanate	2346	
	Carpaine	442630	
	Quercetin-3-O-glucopyranoside	5280804	
	Kaempferol 3-O- rutinoside	5318767	
	Oleic acid	445639	
	Quercetin-3-O-rutinoside	5280805	
	5, 7 Dimethoxycoumarin	2775	
	Protocatechuic acid	72	

	Ferulic acid	445858	
	Beta- carotene	5280489	
	p – Coumaric acid	637542	
	Lycopene	446925	
	Vanillic acid	8468	
	Beta- cryptoxanthin	5281235	
	Chlorogenic acid	1794427	
	Isochlorogenic acid	6436237	
	Sinapic acid	637775	
	Malic acid	525	
	Coumaric acid	323	
	Cinnamic acid	444539	
4. **Momordica charantia**	Daucosterol	5742590	[93]
	Campesterol	173183	
	Stigmasterol	5280794	
	Δ5–avenasterol	5281328	
	Clerosterol	5283638	
	Diosgenin	99474	
	25-dimethoxycucurbita-6	44445558	
	25,26-dihydroelasterol	22295562	

	Palmitic acid	985	
	Myristic acid	11005	
	Pentadecanoic acid	13849	
	Arachidic acid	10467	
	Stearic acid	5281	
	Oleic acid	445639	
	Linoleic acid	5280450	
	Capric acid	2969	
	Lauric acid	3893	
	Docosanoic acid	8215	
	Heneicosanoic acid	16898	
	Nonadecanoic acid	12591	
	Tridecanoic acid	12530	
	Heptadecanoic acid	10465	
	Tetracosanoic acid	11197	
	Gallic acid	370	
	Chlorogenic acid	1794427	
	Caffeic acid	689043	
	Gentisic acid	3469	
	Homogentisic acid	780	

	Epicatechin	72276	
	Isoquercitrin	5280804	
	Gallocatechin gallate (GCG)	5276890	
	Naringin	442428	
	Myricetin	5281672	
	3-coumaric acid	637541	
	Ellagic acid	5281855	
	Ferulic acid	445858	
	Protocatechuic acid	72	
	Salicyclic acid	338	
	Vanilic acid	8468	
	Apigenin	5280443	
	Biochanin A	5280373	
	Epigallocatechin	72277	
	Luteolin	5280445	
	β-sitosterol	222284	
	Clerosterol	5283638	
	Arachidic acid	10467	
	palmitoleic acid	445638	
	p-coumaric acid	637542	

	Syringic	10742	
	β-resorcylic acid	9338	
	Veratric acid	7121	
	Gadoleic acid	5282767	[94]
	α-eleostearic acid	5281115	
	Quercitrin	5280459	
	Quercetin	5280343	
	Palmitoleic acid	445638	
	t-cinnamic acid	444539	
	Naringenin	439246	[95]
	Hesperidin	10621	
	Rutin	5280805	
	Catechin	9064	
	Kaempferol	5280863	
5. **Hippophae rahmnoides**	Gallic acid	370	[96]
	Chlorogenic acid	1794427	
	Caffeic acid	689043	
	Trans p-cumaric acid	637542	
	Ferulic acid	445858	
	Quercetin-3-galactoside	5281643	

	Rutin	5280805	
	Myricetin	5281672	
	Quercitrin	5280459	
	Quercetin	5280343	
	Luteolin	5280445	
	Vitexin	5280441	
	Kaempferol	5280863	
	Luteolin-7-glucoside	5280637	
	Lutein	5281243	
	Zeaxanthin	5280899	
	β-Carotene	5280489	
	Isorhamnetin	5281654	[97]
	Quercetin	5280343	
	Quercetin-3-methylether	5280681	
	Pentamethylquercetin	97332	
	Luteolin	5280445	
	Syringetin	5281953	
	Kaempferol	5280863	
	Naringenin	439246	
	Catechin	9064	

	Epicatechin	72276	
	Epigallocatechin	72277	
	Epicatechin gallate	107905	
	Gallocatechin	65084	
	Gallocatechin gallate	5276890	
	Leucocyanidin	71629	
	Proanthocyanidin	108065	
	Isorhamnetin-3-O-rutinoside	5481663	
	Isorhamnetin-3-O-glucoside	5318645	
	Isorhamnetin-3-O-rhamnoside	23634491	
	Isorhamnetin-3-O-neohesperidoside	24204448	
	Isorhamnetin-3-O-glucoside-7-O-rhamnoside	72188972	
	Isorhamnetin-3,7-O-diglucoside	5323537	
	Isorhamnetin-3-O-galactoside	5318644	
	Quercetin-3-O-glucoside	5280804	
	Naringin	442428	
	Quercetin-7-O-glucoside	5381351	
	Quercetin-3-O-rhamnoside	5280459	
	Quercetin-3-O-hexoside	5378597	
	Quercetin-3-O-galactoside	5281643	

	Quercetin-3-O-rhamnosylglucoside	5748363	
	Quercetin-3-O-sophoroside-7-O-rhamnoside	102085574	
	Quercetin-3-O-galactoside-7-O-rhamnosid	14130922	
	Quercetin-3-O-glucoside-7-O-rhamnoside	49842399	
	Syringetin-3-O-rutinoside	102402404	
	Kaempferol-3-O-rutinoside	5318767	
	Kaempferol-3-O-glucoside	5282102	
	Kaempferol-3-O-neohesperidoside	5318761	
	Kaempferol-3-O-sophoroside-7-O-rhamnoside	102004842	
	Delphinidin-3-glucoside	165558	
	Cyanidin-3-galactoside	10299753	
	Delphinidin-3-rutinoside	192918	
	Cyanidin-3-glucoside	197081	
	Cyanidin-3-rutinoside	441674	
	Malvidin-3-glucoside	443652	
	Peonidin-3-glucoside	443654	
	Peonidin-3-arabinoside	91810651	
	Malvidin-3-galactoside	5484292	
	Tiliroside	5320686	
	Flavonolignans	341092908	

	Phytosterols	12303662	
	Quinic acid	6508	[98]
	Kaempferol-3-sophorotrioside-7-rhamnoside	44258849	
	Kaempferol-3-sophoroside-7-rhamnoside	102004842	
	Isorhamnetin-3-sophoroside-7-rhamnoside	74978261	
	Kaempferol-3-glucoside-7-rhamnoside	6451393	
	Malic acid	525	
	Citric acid	311	
6. **Euphorbia hirta**	Benzoic acid	243	[99]
	Benzamide	2331	
	Gallic acid	370	
	Ethyl gallate	13250	
	Methyl gallate	7428	
	Protocatechuic acid (PCA)	72	
	4-ethenyl-2-methoxyphenol (4-vinylguaiacol)	332	
	1,2-benzenedicarboxylic acid,diisooctyl ester (diisooctyl phthalate)	33934	
	Tetradecane	12389	
	Isolintetralin	101241675	
	Lintetralin	11361584	

	Virgatusin	10549813	
	Urinaligran	637287	
	7-hydroxyhinokinin	100935372	
	Pinoresinol	73399	
	Syringaresinol	100067	
	Syringaresinol diglucoside	226371	
	Pinoresinol glucoside	11168362	
	3,5-O-dicaffeoylquinic acid	6474310	
	Ethyloctadecanoate	8122	
	Geranyl linoleate	91694960	
	Oleic acid	445639	
	Pentadecanoic acid	13849	
	Tetradecanoic acid	11005	
	Bumaldoside A	102163395	
	Byzantionoside B	14135395	
	Corchoionoside C	10317980	
	Roseoside	9930064	
	Geranyl acetate	1549026	
	Neryl acetate	1549025	
	Hexadecanal	984	

	Tetradecanamide	69492	
	Methyl linolenate	5319706	
	Methyl linoleate	5284421	
	Glyceryl monolinoleate	5283469	
	Ethyl linoleate	5282184	
	Linolenic acid	5280934	
	Linoleic acid	5280450	
	2-monopalmitin	123409	
	2-monostearin	79075	
	Triolein	5497163	
	Phytol	5280435	
	Gibberellin	6466	
	Ponicidin	92043456	
	Albopilosin H	11566162	
	kaur-16-ene	10901750	
	Citronellol	8842	
	Camphol	6552009	
	Gamma-tocopherol	92729	
	Isospathulenol	14038848	
	Beta-elemene	6918391	

	Neointermedeol	11877394	
	Beta-bisabolene	10104370	
	Cis-nerolidol	5320128	
	Alpha-humulene	5281520	
	Alpha-farnesene	5281516	
	Beta-caryophyllene	5281515	
	Farnesol	3327	
	Neophytadiene	10446	
	Taraxerolacetate	94225	
	Taraxerone	92785	
	Taraxerol	92097	
	Citroside A	14312562	
	Lupeol	259846	
	Friedelin	91472	
	Alpha-amyrin	73170	
	Beta-amyrin	73145	
	Cycloartenol	92110	
	Campesterol	173183	
	Stigmasterol	5280794	
	Gamma-sitosterol	457801	

	Beta-sitosterol	222284	
	Isojaponin A	101836940	
	Azidocyclohexane	88153	
	Hydroxycitric acid	123908	
	Citric acid	311	
	Malic acid	525	
	3,5-digalloylquinic acid	460896	
	1,5-dibromo-3-methylpentane	138258	
	1-bromo-6-chlorohexane	80516	
	2,3-dihydrobenzofuran	10329	
	3,5-dihydroxy-6-methyl-2,3-dihydropyran-4-one	119838	
	Chelidonic acid	7431	
	1-(3-methyl-3-butenyl)pyrrolidine	558410	
	Nonanenitrile	16715	
	Trigalloylquinic acid	129706725	
	Chlorogenic acid	1794427	
	Quinic acid	6508	
	Shikimic acid	8742	
	Quercitol	441437	
	Ternatoside C	17757758	

	Linocinnamarin	12311284	
	6′-O-galloylsalicin	10741653	
	Syringin	5316860	
	5-hydroxymethyl-2-furancarboxaldehyde	237332	
	Xanthoxylin	66654	
	Megastigmatrienone A	6437599	
7. **Boesenbergia rotunda**	Alpinetin	154279	[100]
	Cardamonin	641785	
	Pinostrobin	73201	
	Pinocembrin	68071	
	GERANIOL	637566	
	Silybin	31553	
	Caffeic acid	689043	
	Coumaric acid	323	
	Hesperidin	10621	
	kaempferol	5280863	
	Naringin	442428	
	Quercetin	5280343	
	Camphor	2537	
	Fenchene	440966	

	Cineole	2758	
	hemanthidine	3002914	
	Limonene	22311	
	Boesenbergin A	6313827	[101]
	(+)-Krachaizin A	11729201	
	Panduratin A	6483648	
	Rotundaflavone Ia	101863268	
	Nerol	643820	
	alpha-Fenchene	28930	
	Chlorogenic acid	1794427	
	Rutin	5280805	[102]
	Ferulic acid	445858	
	Sinapic acid	637775	
	Gallic acid	370	
	Luteolin	5280445	
	Diosmin	5281613	
8. **Cymbopogon citratus**	P-coumaric acid	637542	[103]
	Luteolin	5280445	
	Kaempferol	5280863	
	Caffeic acid	689043	

	Catechol	289	
	Hydroquinone	785	
	Geranial	638011	
	Isopulegol	24585	
	Anthraquinone	6780	
	Isoorientin	114776	[104]
	Isoscoparin	442611	
	Orientin	5281675	
	Chlorogenic acid	1794427	
	Nerolidol	8888	[105]
	B-Elemene	6918391	
	B-Eudesmol	91457	
	Myrtenal	61130	
	Piperitone	6987	
	a-Cubebene	442359	
	Elemol	92138	
	Humulene	5281520	
	Caryophyllene	5281515	
	Cubenol	11770062	
	Cubebol	11276107	
	Carvone	7439	

	Nonanenitrile	16715	
	8-Prenylnaringenin	480764	[106]
	Aspilactonol B	139586885	
	12-Oxo-phytodienoic acid	5280411	
	3-Hydroxyglabrol	480854	
	Amurensin	5318156	
	13-Hydroxynorjavanicin	102514930	
	Citral	638011	
	2-Acetylcyclopentanone	98471	[107]
	Cyclooctane	9266	
	Furan-2-carbohydrazide	18731	
	Epoxy-linalooloxide	537453	
	2-Methoxy-4-vinylphenol	332	
	Triallylsilane	6329064	
	Geranyl acetate	1549026	
	Vanillin	1183	
	Adamantane	9238	
	Heptadecanoic acid	10465	
	Phytol	5280435	
	Campesterol	173183	

	Stigmasterol	5280794	
	Eicosane	8222	
9. **Uncaria tomentosa**	Ajmalicine	441975	[108]
	Campesterol	173183	
	Akuammigine	1268096	
	Sitosterols	3084097	
	Rutin	5280805	
	chlorogenic acid	1794427	
	Speciophylline	168985	
	cinchonain	442675	
	Corynoxeine	44568160	
	Harman	5281404	
	Epicatechin	72276	
	Hirsuteine	3037151	
	Corynantheine	3037997	
	Hirsutine	3037884	
	loganic acid	89640	
	Mitraphylline	94160	
	Isocalamendiol	12302240	
	oleanolic acid	10494	

	ursolic acid	64945	
	Lyaloside	11092621	
	Rhynchophylline	5281408	
	palmitoleic acid	445638	
	procyanidins	107876	
	Stigmasterol	5280794	
	vaccenic acid	5281127	
	rotundifoline	139055911	
	quinovic acid	120678	
	Uncarine F	12304288	
	Speciophylline	168985	
	Mitraphylline	94160	
	Isomitraphylline	11726520	
	Pteropodine	10429112	
	Isopteropodine	9885603	
	Corynoxeine	44568160	
	Rhynchophylline	5281408	
10. **Houttuynia cordata**	Gallic acid	370	[109]
	Chlorogenic acid	1794427	
	Vanillic acid	8468	

	Syringic acid	10742	
	Sinapinic acid	637775	
	Rutin	5280805	
	Quercetin	5280343	
	Decanal	8175	[110]
	Camphene	6616	
	bornyl acetate	6448	
	α-pinene	6654	
	Limonene	22311	
	4-terpineol	11230	
	linalool	6549	
	Afzelin	5316673	
	Quercitin	5280343	
	Isoquercitin	10813969	
	Apigenin	5280443	
	Kaempferol	5280863	
	Isorhamnetin	5281654	
	Phloridzin	6072	
	Avicularin	5490064	
	Cryptochlorogenic Acid	9798666	

	Neochlorogenic acid	5280633	
	Quinic acid	6508	
	Caffeic Acid	689043	
	Methyl vanillate	19844	
	Vanillin	1183	
	Stigmast-4-en-3-one	5484202	
	stigmast-4-ene-3,6- dione	5490007	
	Alanine	5950	
	Proline	145742	
	Lysine	5962	
	Aspartic acid	5960	
	methionine	6137	
	tryptophane	6305	
	glutamic acid	33032	
	leucine	6106	
	Threonine	6288	
11. **Lippia alba**	α-Pinene	6654	[111]
	Sabinene	10887971	
	6-Methyl-5-hepten-2-one	9862	
	Myrcene	31253	

	Limonene	22311	
	Linalool	6549	
	Trans-sabinol	6429076	
	Myrtenyl acetate	61262	
	Carvone	7439	
	Geraniol	637566	
	Geranyl acetate	1549026	
	α-Guaiene	5317844	
	Myricetin	5281672	
	Cubebol	11276107	
	Citral	638011	[112]
	germacrene D	5317570	
	α-muurolene	12306047	
	α-cubenene	442359	
	patchoulane	29408	
	2-ethyl-*p*-xylene	15653	
	Terpinolene	11463	[113]
	Borneol	1201518	
	Longicyclene	564934	
	Longifolene	289151	

	Sesquiterpenes	667450	
	allo-aromadendrene	42608158	
	geranyl formate	5282109	
	α-terpinene	7462	
12. **Lippia citriodora**	α-Pinene	6654	[114]
	Sabinene	18818	
	1-Octen−3-ol	18827	
	6-methyl−5-Hepten−2-one	9862	
	Myrcene	31253	
	α-Terpinene	7462	
	p-Cymene	7463	
	Limonene	22311	
	Linalool	6549	
	Perillene	68316	
	Citronellal	7794	
	Terpinen−4-ol	11230	
	α-Terpineol	17100	
	trans-Carveol	94221	
	Nerol	643820	

	Carvone	7439	
	Geraniol	637566	
	α-Copaene	12303902	
	Geranyl acetate	1549026	
	Allo-Aromadendrene	42608158	
	ar-Curcumene	3083834	
	Spathulenol	92231	
	α-Cadinol	10398656	
	β-bourbonene	62566	[115]
	Nonanal	31289	
	Verbenol	61126	
	Bicyclogermacrene	13894537	
	Furfural	7362	
	Bergamotene	521569	
	α-Cedrene	6431015	
	Santonin	221071	
	Fluoranthene	9154	
	α-Cubebene	442359	
	Zingiberene	92776	
	Aromandendrene	91354	

	Carvacrol	10364	
	Farnesol	3327	
13. **Leucaena leucocephala**	Tetratetracontane	44379994	[116]
	Oxalic acid, allyl hexadecyl ester	6420236	
	Squalene	638072	
	Octacosane	12408	
	Hexatriacontane	12412	
	5-Octadecene	5364598	
	1-Octadecyne	69425	
	3,7,11,15-Tetramethyl-2-hexadecen-1-ol	5366244	
	Pentadecanoic acid, 14-methyl-, methyl ester	21205	
	9,12-Octadecadienoic acid, methyl ester	5284421	
	Hexadecanoic acid, 15-methyl-, methyl ester	522345	
	9,12,15-Octadecatrienoic acid, methyl ester	5367462	
	3,7,11-Tridecatrienenitrile, 4,8,12-trimethyl	5365886	
	2-Dodecene	5352911	
	7-Hexadecene	520743	
	5-Eicosene	5364600	
	1-Docosene	74138	
	Heptacosanoic acid, methyl ester	41517	
	N-Hexadecanoic acid	985	
	Betulin	72326	[117]

	Betamethasone	9782	
14. **Quercus lusitanica**	Cinnamic acid	445858	[118], [119], [120], [121]
	Gallotannin	250395	
	Ellagitannin	44584733	
	Triterpenes	451674	
	Ellagic acid	5281855	
	Chlorogenic acid	1794427	
	Benzoic acid	243	
	Chlorocatechin	22297292	
	Polydatin	5281718	
	Quercetin	5280343	
	Pyrocatechol	289	
	Gallic acid	370	
	Catechin	9064	
	4-Hydroxybenzoic acid	135	
	Pyrogallol	1057	
	Naringin	442428	
	Rutin	5280805	
	Syringic acid	10742	
	1,8–cineol	2758	
	Sesquiterpenoids	11703004	
	Caffeic acid	689043	
	3,4-Dihydroxybenzoic acid	72	
	Epicatechin	72276	
	Epigallocatechin	72277	
	Kaempferol	5280863	
	Glucopyranoside	5793	
	Proanthocyanidin	122738	

	Vescalagin	168165	
	Grandinin	492392	
	Castalagin	168165	
	Phloroglucinol dihydrate	80196	
	4-propylresorcinol	87874	
	Vanillin	1183	
	Coniferaldehyde	5280536	
	Acetovanillone	2214	
	Syringaldehyde	8655	
	Anthraquinones	6780	
	Methyl-oleanolate	92900	
	Nyctanthic acid	12313631	
	Methyl-betulate	423298	
	Roburin E	101670389	
	2,5-Dihydroxybenzoic acid	3469	
	Quinic acid	6508	
	Myricetin	5281672	
	Apigenin	5280443	
	Rosmarinic acid	5281792	
	Nyctanthic acid	12313631	
	Cis-p-mentha -1(7),8-dien-2-ol	6429040	
	3-Nonynoic acid	534236	
	3-Trifluoroacetoxypentadecane	534406	
	Pterin -6-carboxylic acid	135403803	
	Ethyl iso-allocholate	6452096	
	Benzene-1,2,3-triol	1057	
15. **Psidium guajava**	Eucalyptol	2758	[122]
	α-Terpineol	17100	

	Pyrogallol	1057	
	Caryophyllene	5281515	
	Aromadendrene	11095734	
	Cadina-3,5-diene	91723677	
	α- Humulene	5281520	
	Alloaromadendrene	10899740	
	Nerolidol	5284507	
	(-)-Globulol	12304985	
	Viridiflorol	11996452	
	Myricetin	5281672	
	Luteolin-7-O-glucuronide	13607752	
	Quercetin	5280343	
	Catechin	9064	
	Quinic acid	6508	
	Epicubenol	12046149	
16. **Azadirachta indica**	dl-Homoserine	779	[123]
	2-Furanmethanol	7361	
	2-Cyclopentene-1,4-dione	70258	
	Propanoic acid, 2-hydroxy-2-methyl-	11671	
	Butanoic acid, 4-hydroxy-	10413	
	(+-)-4-Amino-4,5-dihydro-2(3H)-furanone	538736	
	1-Pyrrolidineethanamine	1344	
	2,4-Dihydroxy-2,5-dimethyl-3(2h)-furan-3-one	538757	
	Glycerin	753	
	4(H)-Pyridine, N-acetyl –	556800	
	Aziridine, 2-isopropyl-1,3-dimethyl-, trans-	535823	
	Butane, 1-(ethenyloxy)-3-methyl-	142381	
	2,3-Pentanedione, 4-methyl-	24115	

	Butanamide	10927	
	1,3,5-Triazine-2,4,6-triamine	7955	
	4-(4-Methyl-piperazin-1-yl)-1,5-dihydro-imidazol-2-one-	550632	
	Acetone, 1-[4-(dimethylaminoethoxy)phenyl]-	547011	
	3-Amino-2-oxazolidinone	65725	
	4H-Pyran-4-one, 2,3-dihydro-3,5-dihydroxy-6- methyl-	119838	
	N-Methylpyrrole-2-carboxylic	81453	
	4H-Pyran-4-one, 3,5-dihydroxy-2-methyl-	70627	
	Proline, N-methyl-, butyl ester	527835	
	L-Proline, 1-methyl-5-oxo-, methyl ester	12361160	
	2-Methoxy-4-vinylphenol	332	
	Phenol, 2,6-dimethoxy-	7041	
	Phenol, 2-methoxy-3-(2- propenyl)-	596373	
	1,5-Diazabicyclo[4.4.0]dec-5-en-2-one	6424223	
	4-Isopropenyl-4,7-dimethyl-1-oxaspiro[2.5]octane	543441	
	2(4H)-Benzofuranone, 5,6,7,7a-tetrahydro-4,4,7a-trimethyl-	27209	
	Ethanone, 1-(3,4-dimethoxyphenyl)	14328	
	(7,7-Dimethyl-2-oxobicyclo[2.2.1]hept-1-yl) methanesulfonic acid, methyl ester	573022	
	Ethyl N-(2-methylphenyl) carbamate	239624	
	Quinoline, 2-ethyl-	137113	
	2-Butynoic acid, 4-cyclohexyl-4-oxo-, ethyl ester	543518	
	5-Isopropenyl-1,2-dimethylcyclohex-2-enol	536558	
	(4,4-Dimethyl-2,4,5,6-tetrahydro-1H-inden-2- yl)acetic acid	590124	
	3-Buten-2-one, 4-(4-hydroxy- 2,2,6-trimethyl-7-oxabicyclo[4.1.0]hept-1-yl)-	5371267	

	1,4-Benzenediol, 2,6-dimethyl-	69560	
	5-Isopropyl-3,3-dimethyl-2-methylene-2,3- dihydrofuran	586164	
	4-((1E)-3-Hydroxy-1-propenyl)-2-methoxyphenol	1549095	
	5-Bromopentanoic acid, 2-isopropoxyphenyl ester	573465	
	3,7,11,15-Tetramethyl-2-hexadecen-1-ol	5280435	
	4-Decenoic acid, 3-methyl-, (E)-	5371379	
	à-D-Glucopyranoside, à-D-glucopyranosyl	5988	
	3-Heptanol, 3,5-dimethyl-	140546	
	Bicyclo[4.4.0]dec-2-ene-4-ol, 2-methyl-9- (prop-1-en-3-ol-2-yl)-	535256	
	Cholesta-4,6-dien-3-ol, (3á)-	33010	
	-Nimocinolide	6442906	[124]
	3-Deacetylsalannin	6450192	
	3beta-Hydroxy-20(29)-lupene	16219576	
	6-Deacetylnimbinene	102285347	
	Azadirachtanin	102146586	
	Acetylneotrichilenone	298061	
	Desacetylnimbinolide	102285346	
	Epoxyazadiradione	122801	
	Gedunin	114923	
	melianin B	101650342	
	Nimbocinolide	13875774	
	Phytosterols	12303662	

	Nimbolin A	101650373	
17. **Ocimum sanctum**	Eugenol (1-hydroxy-2-methoxy-4-allylbenzene)	3314	[125]
	Urosolic acid (2,3,4,5,6,6a, 7,8,8a,1O,1l,12,13, 14btetradecahydro-1H-picene-4a-carboxylic acid)	220774	
	Carvacrol (5-isopropyl-2-methylphenol)	10364	
	Linalool (3,7-dimethylocta-1,6-dien-3-ol)	6549	
	Caryophylline (4,1l,1l-trimethyl-8-methylene-bicyclo[7.2.0] undec-4-ene)	5281515	
	Estragol (1-allyl-4-methoxybenzene)	8815	
	Rosmarinic acid	5281792	
	Cirsimaritin (5,4'-dihydroxy-6, 7-dimethoxyflavone)	188323	
	Apigenin	5280443	
	alpha-Cadinol	10398656	[126]
	Caryophyllene oxide	174221	
	T-Cadinol	160799	
	Spathulenol	92231	
	Viridiflorol	11996452	
	Champhene	6616	
	Limonene	22311	
	Camphene hydrate	101680	
	Carene	26049	
	TERPINOLENE	11463	
	Sabinene hydrate	62367	
	Ocimene	5281553	
	Myrcene	31253	
	Sabinene	18818	
	Tricyclene	79035	

	Borneol	64685	
	Methyl Eugenol	7127	
	1,8-Cineole	2758	
	Geraniol	637566	
	trans-Linalool oxide (furanoid)	6432254	
	Terpinen-4-ol	11230	
	Camphor	2537	
	Fenchone	14525	
	trans-Sabinene hydrate	6431628	
	p-Methoxy cinnamaldehyde	641294	
	Cubenol	11770062	
