# Supplementary material for: Identification of Potential Inhibitors Targeting Non-Structural Proteins NS3 and NS5 of Dengue Virus Using Docking and Deep Learning Approaches
Source: Pharmaceuticals (Basel). 2025 Apr 13;18(4):566. doi: 10.3390/ph18040566 (PMC12030398; doi:10.3390/ph18040566)
Supplement: Supplementary file 1 [file pharmaceuticals-18-00566-s001.zip › Table S3, Figure S1, S2.pdf]

Supplementary files

Table S3: Binding Affinity (kcal/mol) and Predicted pIC50 score of the phytochemicals for NS3 and NS5 protein as predicted by AutoDock Vina.

Sl. No.	Ligands Name	PubChem CID	Binding affinity	Predicted pIC50 score
**Phytochemicals for NS3 protein**				
1	DIOSGENIN	99474	-9.4	4.54
2	(-)-Cinchonain IA; Cinchonain 1a	442675	-8.6	4.16
3	Stigmast-4-ene-3,6-dione; 23670-94-2	5490007	-8.5	4.25
4	Ternatoside C	17757758	-8.5	4.58
5	Dehydrocarpaine II	131750992	-8.4	3.96
6	3-Hydroxyglabrol	480854	-8.4	4.80
7	Sitostenone	5484202	-8.1	4.08
8	silibinin	31553	-8.5	4.76
9	Rubiadin 1-methyl ether	96191	-8.3	4.67
10	Rubiadin	124062	-8.3	4.70
11	ellagic acid	5281855	-8.2	4.35
12	Taraxerone	92785	-8.2	4.07
13	Taraxerol	92097	-8.1	4.07
14	ANTHRAQUINONE	6780	-8.0	4.33
15	STIGMASTEROL	5280794	-8.0	4.14
**Phytochemicals for NS5 protein**				
1	Bisandrographolide A	12000062	-10.1	4.43
2	Taraxerol	92097	-10.1	4.07
3	Taraxerone	92785	-9.9	4.07
4	Cycloeucalenol	101690	-9.7	3.93
5	Friedelan-3-one	91472	-9.7	3.61
6	24-Methylenecycloartanol	94204	-9.9	3.93
7	Dehydrocarpaine II	131750992	-9.5	3.96
8	Carpaine	442630	-9.1	4.23
9	NSC 640467	122801	-9.3	4.71
10	beta-Amyrin	73145	-9.3	4.04
11	DIOSGENIN	99474	-9.3	4.54
12	oleanoic acid	485707	-9.1	4.42
13	OLEANOLIC ACID	10494	-8.9	4.42
14	Andrographidin A	13963762	-8.9	4.84
15	Methyl oleanolate	92900	-8.8	4.46

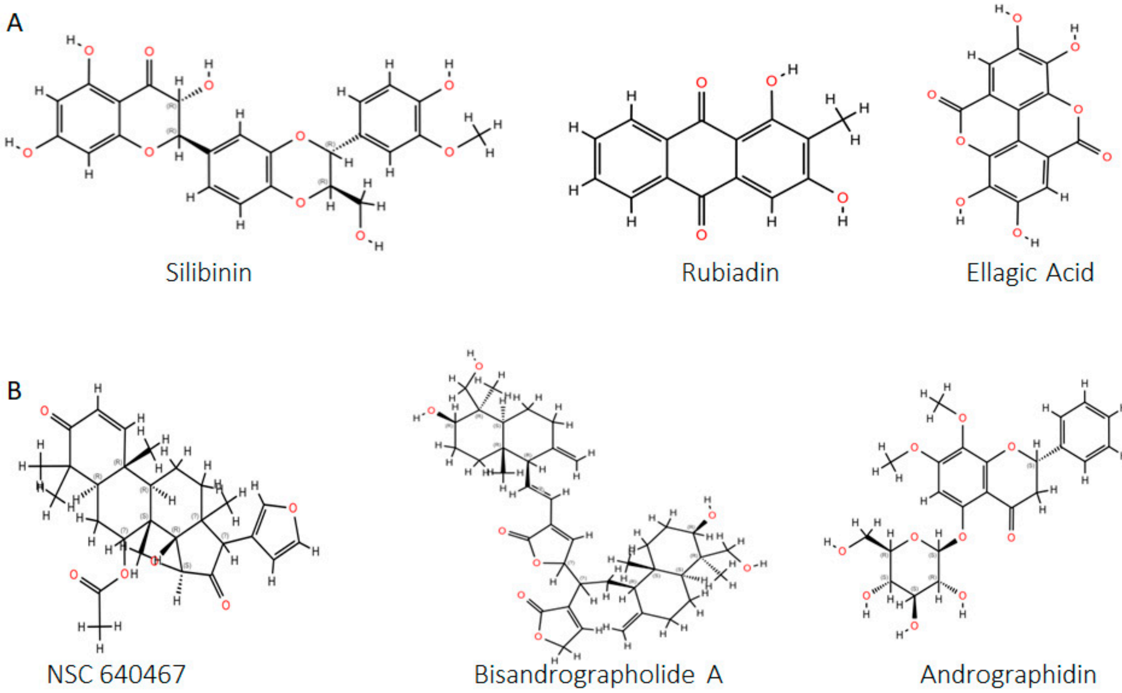

Figure S1. The 2D chemical structures of (A) Silibinin, Rubiadin, and Ellagic Acid, and (B) NSC 640467, Bisandrographolide A, and Andrographidin were created using the 2D Sketcher (Beta) software.

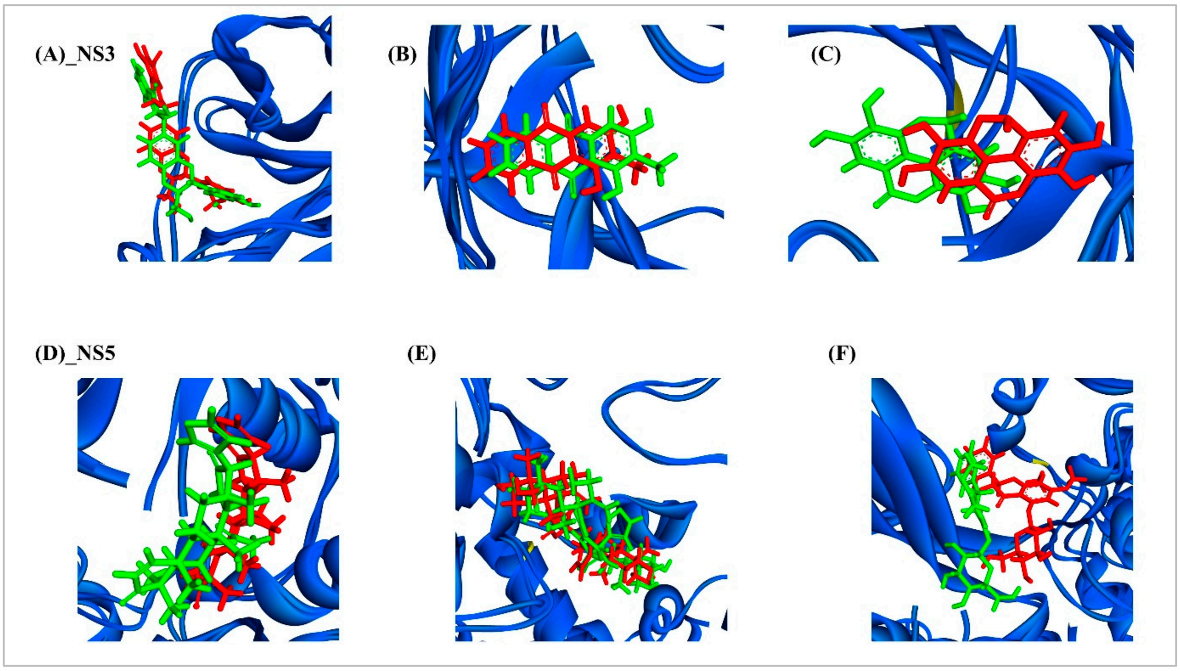

Figure S2: 3D view of the best re-docking pose and conformational superimposition of all lead compounds and their RMSD scores: (A) Silibinin, RMSD value 1.441 Å, (B) Rubiadin, RMSD value 1.515 Å, and (C) Ellagic Acid, RMSD value 1.735 Å for NS3 protein. (D) NSC 640467, RMSD value 1.604 Å, (E) Bisandrographolide A, RMSD value 1.932 Å, and (F) Andrographidin A, RMSD value 2.011 Å, for NS5 Protein. The red color denotes the original crystallographic pose, and the green color denotes the docked pose.

The conformational superimposition and 3D visualization of the top re-docked poses with their respective co-crystallized ligands further confirmed the reliability of our docking protocol. For the NS3 protein, the calculated RMSD values between the docked and reference poses were 1.441 Å for Silibinin, 1.515 Å for Rubiadin, and 1.735 Å for Ellagic Acid. In the case of the NS5 protein, RMSD values were 1.604 Å for NSC 640467, 1.932 Å for Bisandrographolide A, and 2.011 Å for Andrographidin A. These values, all near or below the widely accepted threshold of 2.0 Å, demonstrate that the docking method was capable of accurately reproducing the experimentally observed binding conformations. The close structural alignment of docked and crystallographic poses supports the robustness and predictive reliability of our docking workflow
